# SUBSIDIZED SENIOR HOUSINGS (SSH) FOR LOW-INCOME OLDER ADULTS IN THE U.S.: A SCOPING REVIEW

**DOI:** 10.1093/geroni/igac059.2768

**Published:** 2022-12-20

**Authors:** Sojung Park, Soobin Park, Takashi Amano, Jihye Baek, BoRin Kim, Byeongju Ryu

**Affiliations:** Washington University in Saint Louis, Saint Louis, Missouri, United States; Washington University in St. Louis, Saint Louis, Missouri, United States; Rutgers University-Newark, Newark, New Jersey, United States; Washington University in St. Louis, St. Louis, Missouri, United States; University of New Hampshire, Durham, New Hampshire, United States; Washington University in St. Louis, St. Louis, Missouri, United States

## Abstract

Subsidized senior housing (SSH) has been advocated as a key component of a community-based, long-term care policy for low-income older adults that links housing with health and social services to support aging-in-place (AIP). Relative to the accumulating evidence on for-profit housing models, SSH remained understudied. Guided by the Person-Environmental fit perspective, this scoping review described and synthesized existing literature on SSH to identify strengths and gaps in the literature. With a five-step scoping review method, we focused on the empirical studies published from 2010 in the U.S using four electronic databases and additional manual searching. Sixty-six articles met the inclusion criteria. Study participants were predominantly non-Hispanic White and female; most studies were quantitative surveys with a cross-sectional design. Five areas of outcomes emerged: (1) Health and well-being (45%), (2) Healthcare use and health behavior (24%), (3) Social Relations (11%), (4) Housing Relocation (12%), and (5) Technology (8%). In examining the study outcomes, the existing articles primarily focus on the personal (29%) and environmental (71%) dimensions, including 9% of the articles investigating both dimensions. For each dimension, the majority of the research tends to highlight residents’ health conditions and characteristics of supports and service programs in the housing. The largest proportion of the literature studied the relationship between residents’ health and the outcomes related to health and well-being. Future research needs to examine subpopulations of older residents in SSH (e.g., racial/ethnic minority, LGBTQIA, and others) as well as the current and future role of technology in SSH and community partnership.

